# Matrix Metalloproteinase-2 Deficiency Impairs Eye Development in Crispant Zebrafish

**DOI:** 10.3390/cells15211993

**Published:** 2026-10-31

**Authors:** Chenhui Ren, Lu Wei

**Affiliations:** Hainan Province Key Laboratory of One Health, School of Life and Health Sciences, Collaborative Innovation Center of Life and Health, Hainan University, Haikou 570228, China

**Keywords:** eye development, Mmp2, photoreceptor, apoptosis, transcriptomics, zebrafish

## Abstract

Matrix metalloproteinase-2 (Mmp2, also known as gelatinase A) is a zinc-dependent endopeptidase, and mutations in human MMP2 can lead to some human diseases. However, the underlying function and mechanism of *mmp2* during eye development are still unclear. The present study generated *mmp2* crispant zebrafish by CRISPR/Cas9 system. Whole-mount in situ hybridization (WISH), immunofluorescence, qRT-PCR, and transcriptomic and metabolomic analyses were performed. Results showed that *mmp2* knockdown led to small eyes at 48–96 hpf (hours post-fertilization). WISH and qRT-PCR showed that *mmp2* knockdown at 72 hpf (hours post-fertilization) significantly decreased the expression of cone-specific markers (*opn1sw1*, *opn1sw2*, *opn1mw1*, and *opn1lw1*), the rod-specific marker rhodopsin, retinal cell-specific markers (*gnat1* and *gnat2*), and lens-specific markers (*crybgx* and *crygm2c*) in the retina. Immunofluorescence showed increased apoptosis (TUNEL^+^ cells), ectopic localization of the neuronal differentiation marker HuC/D at the ciliary marginal zone, and decreased photoreceptor cells (Zpr1) and photoreceptor (RCVRN) fluorescence. Transcriptomic analysis indicated that 520 differentially expressed genes were enriched in phototransduction and retinol metabolism. Metabolomics analysis indicated that *mmp2* knockdown led to abnormal lipid metabolism and amino acid metabolism. Integrated omics analysis revealed shared pathways involving PPAR signaling and nucleotide sugar metabolism, while correlation analysis highlighted strong associations between eye-development-related genes (e.g., *gnat1*, *gnat2*, *opn1sw1*) and specific metabolites such as 2-palmitoyl-rac-glycerol and 1-hexadecyl-sn-glycero-3-phosphocholine. Collectively, these findings demonstrate that Mmp2 is essential for normal eye development in zebrafish, and its deficiency disrupts retinal cell survival, differentiation, and metabolic homeostasis, providing new insights into the molecular mechanisms underlying Mmp2-associated ocular abnormalities.

## 1. Introduction

Eye malformations, including microphthalmia (a congenitally small eye), coloboma and anophthalmia, represent severe structural birth defects that collectively account for approximately 10–11% of global childhood blindness cases [1,2]. Microphthalmia in particular arises from defective embryonic morphogenesis [3]. Yet the underlying molecular determinants remain incompletely understood. Despite advances in genomic screening technologies, over 100 genes have been linked to microphthalmia–anophthalmia–coloboma (MAC) spectrum disorders, but these explain only around one-third of reported clinical cases [4], underscoring a pressing need to identify novel regulators and pathways critical for ocular development.

Matrix metalloproteinase-2 (Mmp2, also known as gelatinase A) is a zinc-dependent endopeptidase that plays a critical role in tissue remodeling, wound healing, angiogenesis, and embryonic development by degrading extracellular matrix (ECM) components such as collagen IV, laminin, and gelatin [5]. During development, Mmp2 participates in dynamic ECM remodeling, regulating cell migration, differentiation, and morphogenesis [5,6]. Kalev-Altman et al. revealed that Mmp2 promotes neural crest cell migration in avian and mammalian embryos [7]. Zhang et al. demonstrated that zebrafish *mmp2* is maternally expressed from the one-cell stage through at least 72 h post-fertilization. Morpholino-mediated knockdown of *mmp2* results in severe axial truncation defects, indicating that Mmp2 is essential for normal embryogenesis, in contrast to murine knockout studies in which Mmp2 appears dispensable for gross development [8]. Using in vitro and in vivo zymography, Crawford and Pilgrim further characterized the ontogeny and spatial regulation of Mmp activity in the zebrafish embryo, revealing that Mmps, including gelatinases, are subject to complex spatiotemporal regulation during morphogenesis [9]. Importantly, Wyatt and Crawford developed a transgenic reporter of endogenous Mmp2 activation and showed that although Mmp2 is expressed relatively ubiquitously, its post-translational proteolytic activation is tightly restricted to specific locations and times, correlating with patterns of active collagen degradation during tail development [5]. This highlights that the biologically relevant activity of Mmp2 is governed predominantly at the post-translational level rather than by transcriptional regulation alone, a principle that also applies to the retinal context [5,9]. However, the contribution of extracellular microenvironmental factors and matrix-remodeling enzymes to eye size determination remains poorly understood.

In the visual system, Mmps, particularly gelatinases Mmp2 but not Mmp9, have been implicated in retinal ganglion cell (RGC) axonal outgrowth and guidance during mouse development [10]. Studies in *Xenopus* and zebrafish demonstrated that Mmp inhibition disrupts retinotectal projections, and genetic knockdown of *mmp14a* (an Mmp2 activator) reduces RGC axon innervation in the optic tectum [11,12]. Furthermore, Mmp2 has been localized to outgrowing RGC axons and growth cones, suggesting an intrinsic function in axonal growth stimulation [13]. In the retina specifically, Mmp2 has been implicated in multiple cell types and processes. Gu et al. showed that silencing *mmp2* and *mmp9* inhibits the invasion and migration of human retinal microvascular endothelial cells, suggesting a role in retinal angiogenesis [14]. Detry et al. demonstrated, through Mmp2-gene ablation in mice and *mmp2* knockdown in zebrafish, that Mmp2 governs lymphatic vessel formation as an interstitial collagenase, further underscoring its importance in vascular development [15]. In the context of retinal glia, Campbell et al. investigated gelatinase activity in the avian retina and found that microglia, Müller glia, and nonastrocytic inner retinal glia regulate dynamic changes in gelatinase activity through TIMP2 and TIMP3, influencing the formation of Müller glia progenitor cells [16]. More recently, Jaroszynska et al. demonstrated that *mmp2* and *mmp9* are required for Müller glia morphogenesis and glia-neuron integration in the zebrafish retina, and that gelatinase activity is essential for maintaining neuronal integrity and visual function in the mature retina [17]. Notably, Janssens et al. had already detected Mmp2 expression in Müller glia and their end feet within the zebrafish eye, a finding that has now been functionally elaborated by these recent studies [12]. Additionally, the regulation of Mmp2 itself is subject to complex signaling inputs; Gao et al. showed that all-trans retinoic acid upregulates Mmp2 expression through retinoic acid receptor beta in retinal pigment epithelial cells, indicating that retinoid signaling may modulate gelatinase activity in the retina [18]. More recently, Mmp2 has been shown to participate in optic fissure fusion by degrading the fissure basement membrane, a process essential for ventral retinal closure [19]. Although Mmp-associated proteolytic activity has been implicated in retinal development [12] and *mmp2* has been shown to contribute to optic fissure fusion [19], the consequences of *mmp2* perturbation for retinal differentiation, apoptosis, and the associated molecular landscape remain incompletely understood. Here, we therefore characterize *mmp2* function during zebrafish retinal development by detecting the expression of cone-specific markers (*opn1sw1*, *opn1sw2*, *opn1mw1*, and *opn1lw1*), the rod-specific marker rhodopsin, retinal cell-type-specific markers (*gnat1* and *gnat2*), and lens-specific markers (*crybgx* and *crygm2c*) in the retina. We further examined apoptosis by immunofluorescence for TUNEL-positive cells, together with neuronal differentiation HuC/D and photoreceptor markers (Zpr1, RCVRN), and integrated transcriptomic and metabolomic analyses to define the associated molecular landscape.

Zebrafish (*Danio rerio*) provide an exceptional vertebrate model for studying eye development [3,20]. In this study, we aimed to characterize Mmp2 function during zebrafish retinal development using CRISPR/Cas9-mediated knockdown, from classic histological analysis to integrated multi-omic approaches. Our data demonstrate that *mmp2* is essential for proper retinal differentiation, photoreceptor differentiation, and cellular homeostasis, and that disruption of *mmp2* triggers microphthalmia via a network involving lipid metabolism, visual transduction, and apoptosis. These findings establish Mmp2 as a critical regulator of retinal development and offer new mechanistic insights into the molecular basis of microphthalmia.

## 2. Materials and Methods

### 2.1. Zebrafish and Embryo Maintenance

AB-strain zebrafish were raised under standard conditions as previously described [20]. All experimental procedures were approved by the Hainan University Institutional Animal Care and Use Committee (HNUAUCC-2025-00062). Fertilized embryos were collected and cultured in E3 medium. At 72 hpf, embryos were anesthetized using MS-222. All staining experiments were performed in the presence of 0.003% 1-phenyl 2-thiourea (PTU).

### 2.2. gRNA Injection and Generation of Crispants

**Figure 1:**
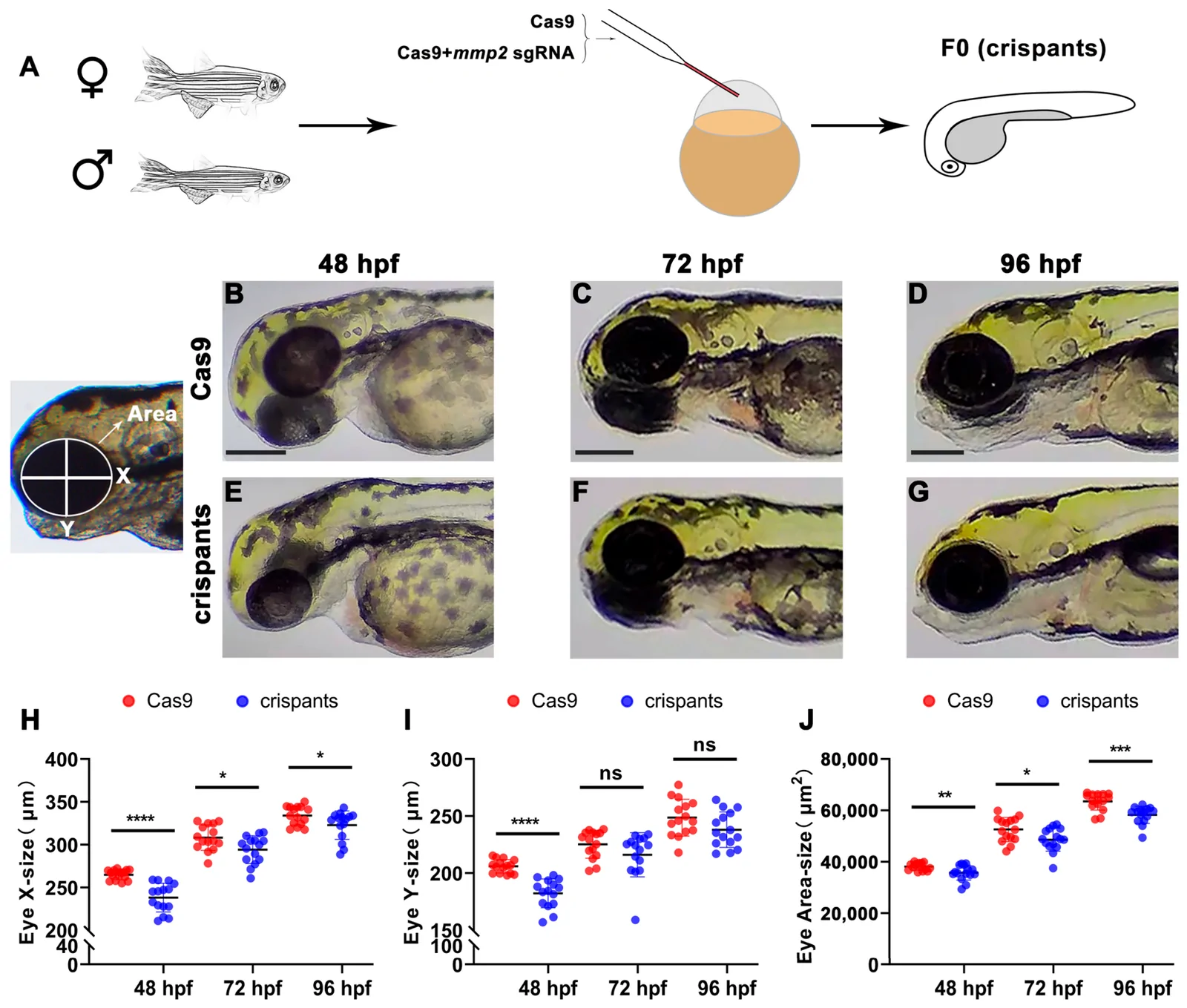
Silencing of *mmp2* induces marked ocular alterations in zebrafish. (**A**) Schematic of the procedure and guide RNAs for generating *mmp2* crispants. (**B**–**G**) Lateral views of zebrafish larvae heads at (**B**,**E**) 48 hpf, (**C**,**F**) 72 hpf, and (**D**,**G**) 96 hpf. The measurement methods for the X-axis and Y-axis of the eyes as well as the area are shown in the left figure. The upper panels (**B**–**D**) represent the Cas9 group, while the lower panels (**E**–**G**) represent the *mmp2* crispants. Scale bar = 200 μm. (**H**–**J**) Measurements of ocular size, including the (**H**) X-axis length, (**I**) Y- axis length and (**J**) area. * *p* < 0.05, ** *p* < 0.01, *** *p* < 0.001, **** *p* < 0.0001, versus control; ns, not significant. *n* = 15 individual larvae per group.

To knock down the *mmp2* gene, multiple guide RNA target sequences were designed within exon 6. Single-guide RNAs (sgRNAs: TACGGATTCTGTCCTGAGAC AGG) were synthesized via PCR amplification followed by in vitro transcription. A mixture of sgRNAs and Cas9 protein was microinjected into zebrafish embryos [21,22], and the injected group was compared to a control group injected with Cas9 only (Figure 1A). At 2 days post-fertilization (dpf), 20 larvae from each group were randomly selected for Sanger sequencing to assess *mmp2* knockdown efficiency (Figure S2A). The sgRNA synthesis and efficiency validation were performed by Hunter Biotech (Hangzhou, China).

### 2.3. Analysis of Eye Morphology

At 48, 72 and 96 hpf, larvae were photographed using a stereo microscope (Motic, Xiamen, China), and eye measurements were taken with ImageJ software (Version 1.53e) [23]. The length of eye parallel to the body axis was defined as the X-axis, the length perpendicular to the X-axis as the Y-axis, and the perimeter was defined as the circular outline [24]. To avoid pseudoreplication, each individual larva was considered as one biological replicate.

### 2.4. Whole-Mount in Situ Hybridization

The plasmids for *opn1mw1*, *crybgx* and *crygm2* were synthesized by Huayu Gene (Wuhan, China). Plasmids for *mmp2*, *rhodopsin*, *gnat1*, *gnat2*, *vsx2*, *opn1sw1*, *opn1sw2* and *opn1lw1* were constructed using PCR with the primer sequences listed in Table S1. All plasmids were linearized and transcribed to generate antisense RNA probes (DIG RNA Labeling Kit, Roche, Basel, Switzerland). Embryos at 48 hpf and 72 hpf were fixed in 4% PFA, subjected to gradient dehydration and rehydration in methanol/PBST, and then digested with protease K. The embryos were incubated in a hybridization buffer containing the corresponding probe, followed by the addition of anti-DIG antibody (Anti-Digoxigenin-AP, Roche, Basel, Switzerland). They were then incubated at 4 °C overnight and subsequently stained using the NBT/BCIP kit (Beyotime, Shanghai, China). Images were captured with a Motic stereomicroscope. The protocol was performed as described [25].

### 2.5. Total RNA Extraction and RT-qPCR

Larvae at 48 and 72 hpf were collected and homogenized on ice. RNA lysate and diluent were added, and the supernatant was collected after centrifugation. Subsequently, 0.5 volumes of anhydrous ethanol were added and the mixture was rapidly vortexed. The mixture was then transferred to a centrifuge tube, and genomic DNA was digested with DNase I to remove it. Finally, total RNA was washed with nuclease-free water for elution (Promega, Beijing, China). The total RNA was reverse transcribed to generate cDNA, which served as a template for real-time fluorescence quantitative PCR to detect the expression levels of the gene (Vazyme, Nanjing, China). The specific primer sequences are provided in Supplementary Table S2. Relative expression was calculated using the 2^−ΔΔCt^ method [26].

### 2.6. Immunofluorescence Staining

At 72 hpf, larvae were fixed overnight in 4% PFA and embedded in paraffin. Eye sections were obtained and processed using in Situ Cell Death Detection Kit (Roche, Basel, Switzerland) according to the manufacturer’s instructions to detect TUNEL signals. For detection of HuC/D, Recoverin and Zpr1, the following primary and secondary antibodies were used: HuC/HuD Monoclonal Antibody (Invitrogen, Carlsbad, CA, USA), Recoverin Polyclonal antibody (Proteintech, Wuhan, China), Anti-Zpr1 antibody (Abcam, Cambridge, UK), Goat anti-Mouse IgG-FITC Antibody (Absin, Shanghai, China), and Goat anti-Rabbit IgG-FITC Antibody (Absin, Shanghai, China). Nuclei were counterstained with DAPI (Biosharp, Hefei, China) after washing. Fluorescence images were acquired on a Leica THUNDER Spinning Disk confocal system (Leica Microsystems, Wetzlar, Germany) equipped with a CrestOptics CICERO spinning disk unit (CrestOptics, Rome, Italy) (pinhole size 50 μm, pitch 250 μm) and an 89 North LDI4 laser launch (89 North, Williston, VT, USA). A 20× air objective (NA 0.40) was used for all imaging. Excitation was performed using the appropriate laser lines (405 nm, 470 nm and 555 nm depending on the fluorophore). Images were acquired at 16-bit depth with a resolution of 1024 × 1024 pixels, and the laser power and detector gain were adjusted for each channel to avoid pixel saturation while maximizing the dynamic range. All images were acquired and processed using the Leica LAS X software (Version 4.5.0.25531).

Fluorescence intensity quantification was performed using ImageJ. The acquired multichannel images were first split into individual single-channel images (Image > Color > Split Channels). For each channel of interest, regions of interest (ROIs) of identical size were manually delineated using the appropriate selection tool. The measurement parameters were defined using the “Analyze > Set Measurements” command, where “Mean gray value” was selected to obtain the average fluorescence intensity per pixel within each ROI. Subsequently, the “Analyze > Measure” command was applied to each ROI to record the mean fluorescence intensity. The output “Mean” value from the Results window was used as the average fluorescence intensity for quantitative analysis. For each experimental condition, measurements were taken from a minimum of 8–10 independent fields of view [27]. To avoid pseudoreplication, each individual larva was considered as one biological replicate.

### 2.7. Transcriptome Analysis

For transcriptome analysis, each treatment was performed with three biological replicates. At 72 hpf, 30 larvae/group were collected, frozen in liquid nitrogen and kept at −80 °C until shipment to Applied Protein Technology for sequencing. Total RNA was isolated from each sample via isopropanol precipitation. Only samples meeting the quality thresholds, yielding ≥ 1 µg of total RNA with an RNA integrity number (RIN) of 4 or higher were subjected to library construction and subsequent Illumina sequencing. After removing low-quality reads, each sample generated approximately 40 to 59 million clean reads, corresponding to 5.87–8.60 Gb of clean bases. The overall sequencing quality was high, with Q20 and Q30 scores exceeding 98.5% and 96.0%, respectively, and an average base error rate of 0.02%. To quantify transcript expression levels, the clean reads were aligned to the reference genome. Differentially expressed genes (DEGs) were identified using the DESeq2 package (version 1.34.0) based on the read counts, applying thresholds of |Log2(fold change)| > 1 and adjusted *p*-value (*p*-adjust) < 0.05. Additionally, FPKM (Fragments Per Kilobase of transcript per Million mapped reads) values were calculated for the visualization of gene expression levels. Subsequently, Gene Ontology (GO) enrichment and Kyoto Encyclopedia of Genes and Genomes (KEGG) pathway enrichment analyses were conducted on the DEGs [28,29]. The raw reads were deposited in the NCBI Sequence Read Archive (SRA) database (PRJNA1484010).

### 2.8. Metabolomics Analysis

Metabolomics sample collection and processing were performed in parallel with transcriptomics. After a slow thaw at 4 °C, the samples were mixed with a pre-cooled 2:2:1 methanol/acetonitrile/water solution and vortexed. Subsequent steps included low-temperature ultrasonication, centrifugation, and vacuum concentration of the supernatant. The residue was then reconstituted in a 1:1 acetonitrile/water solution, vortexed, centrifuged, and the final supernatant was analyzed by liquid chromatography-mass spectrometry (LC-MS). For metabolite selection, an OPLS-DA model was generated to obtain variable importance in projection (VIP) values; differential metabolites were defined as those with VIP > 1, *p* < 0.05 and |Log_2_(fold change)| > 1. To gain insight into the biological pathways of the differential metabolites, GO and KEGG pathway annotation was performed. The metabolomics data generated in this study have been deposited in the EMBL-EBI MetaboLights database under the accession number MTBLS15010 (https://www.ebi.ac.uk/metabolights/MTBLS15010, accessed on 28 October 2026).

### 2.9. Joint Analysis of Transcriptome and Metabolome

For the combined transcriptome–metabolome analysis, differential metabolites were defined as those with *p* < 0.05, VIP > 1, and |Log_2_(FC)| > 1, whereas DEGs were identified using *p*-adjust < 0.05 and |Log_2_(FC)| > 1. Correlation and pathway enrichment analyses were performed on the selected DEGs and differential metabolites. The joint analysis was conducted using the cloud platform provided by Applied Protein Technology.

### 2.10. Statistical Analysis

All experiments were repeated at least three times. Statistical analyses were performed using GraphPad Prism 8.0.2 to evaluate significance. An unpaired two-tailed Student’s *t*-test was used for comparisons between two groups. One-way ANOVA was utilized when there was only one control group. Two-way ANOVA was performed for experimental designs involving two independent variables and their interaction. Significance levels were set at * *p* < 0.05, ** *p* < 0.01, *** *p* < 0.001, **** *p* < 0.0001. Non-significant differences were labeled as ns.

## 3. Results

### 3.1. Silencing of mmp2 Induces Marked Ocular Alterations in Zebrafish

To investigate the spatiotemporal expression of *mmp2* during zebrafish embryogenesis, whole-mount in situ hybridization (WISH) and immunohistochemistry (IHC) were performed. WISH revealed dynamic expression of *mmp2* in the brain, eyes and surrounding tissues (Figure S1(A1–A6)). IHC results were largely consistent with WISH, with additional expression detected in the spine at 48 and 72 hpf (Figure S1(B1–B6)). RT-qPCR analysis showed that *mmp2* expression began to increase at 14 hpf and peaked at 96 hpf (Figure S1C). In adult zebrafish, *mmp2* was relatively highly expressed in the spine, heart, brain and eye (Figure S1D). In addition, RT-qPCR confirmed that *mmp2* crispants led to reduced *mmp2* expression level (Figure S2B).

Next, we further investigated the impact of *mmp2* on eye development. *mmp2* crispant mutants were generated using CRISPR/Cas9. Embryonic eye morphology was assessed through measurements of the X-axis, Y-axis, and whole eye area (Figure 1B–G). Compared with the control group, *mmp2* knockdown resulted in a significant reduction in eye X-axis length (Figure 1H) and eye area (Figure 1J) at 48, 72 and 96 hpf, whereas Y-axis length was significantly decreased only at 48 hpf (Figure 1I). These findings indicate that *mmp2* knockdown leads to microphthalmia phenotypes.

### 3.2. Expression Patterns of Eye Marker Genes in mmp2-Deficient Embryos

**Figure 2:**
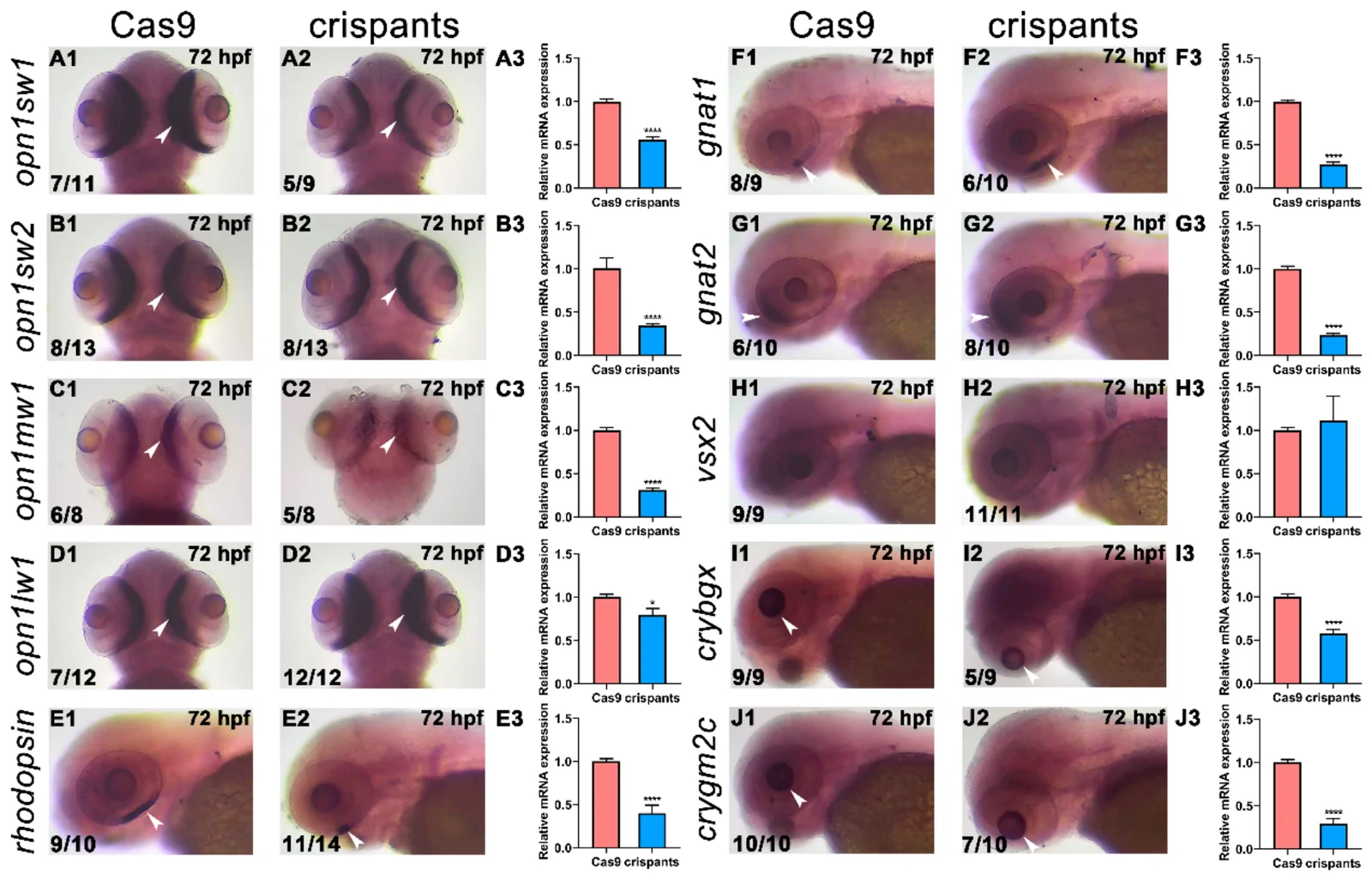
Expression patterns of eye marker genes in *mmp2*-deficient embryos. WISH expression patterns of (**A1**,**A2**) *opn1sw1*, (**B1**,**B2**) *opn1sw2*, (**C1**,**C2**) *opn1mw1*, (**D1**,**D2**) *opn1lw1*, (**E1**,**E2**) *rhodopsin*, (**F1**,**F2**) *gnat1*, (**G1**,**G2**) *gnat2*, (**H1**,**H2**) *vsx2*, (**I1**,**I2**) *crybgx* and (**J1**,**J2**) *crygm2c* in zebrafish embryos at 72 hpf. Arrows indicate the specific regions of gene expression. The numbers in the lower-left corner indicate the number of embryos with the representative phenotype/total number of embryos. (**A3**–**J3**) Relative mRNA expression levels of the corresponding genes quantified by RT-qPCR. * *p* < 0.05, **** *p* < 0.0001, versus control.

To further dissect the role of *mmp2* deficiency in ocular development, we conducted spatial distribution analysis and quantitative expression analysis of genes associated with eye development. At 48 hpf, the expression of cone-specific genes *opn1sw1* (Figure 2(A1,A2) and Figure S3A), *opn1sw2* (Figure 2(B1,B2) and Figure S3B) and *opn1mw1* (Figure 2(C1,C2) and Figure S3C) exhibited no distinct localized pattern; however, at 72 hpf, *mmp2* knockdown led to reduced expression of these genes, consistent with RT-qPCR results. In contrast, *opn1lw1* (Figure 2(D1,D2) and Figure S3D) exhibited an expanded WISH signal although RT-qPCR showed a significant decrease in its overall transcript levels. At both 48 and 72 hpf, *mmp2* knockdown resulted in significantly reduced expression of the rod-specific gene *rhodopsin* (Figure 2(E1,E2) and Figure S3E). The rod transducing α-subunit *gnat1* (Figure 2(F1,F2) and Figure S3F) and the cone cell-specific transducing α-subunit *gnat2* (Figure 2(G1,G2) and Figure S3G) showed no distinct localization at 48 hpf; however, at 72 hpf, *mmp2* deficiency caused a stronger WISH signal for *gnat1* and *gnat2*, whereas RT-qPCR showed extremely significant downregulation. The localization and quantitative expression of the bipolar cell-specific gene *vsx1* in the INL were comparable between the control and *mmp2*-deficient groups (Figure 2(H1,H2) and Figure S3H). The crystallin-specific related genes *crybgx* (Figure 2(I1,I2) and Figure S3I) and *crygm2c* (Figure 2(J1,J2) and Figure S3J) showed distinct lens localization at 48 and 72 hpf, with no significant difference at 48 hpf. At 72 hpf, *mmp2* knockdown led to extra-lens expression of *crybgx* and *crygm2c* and a significant decrease in their expression levels, consistent with RT-qPCR results at 72 hpf.

### 3.3. Mmp2 Deficiency Leads to Enhanced Retinal Cell Apoptosis and Reduced Photoreceptor Cells

**Figure 3:**
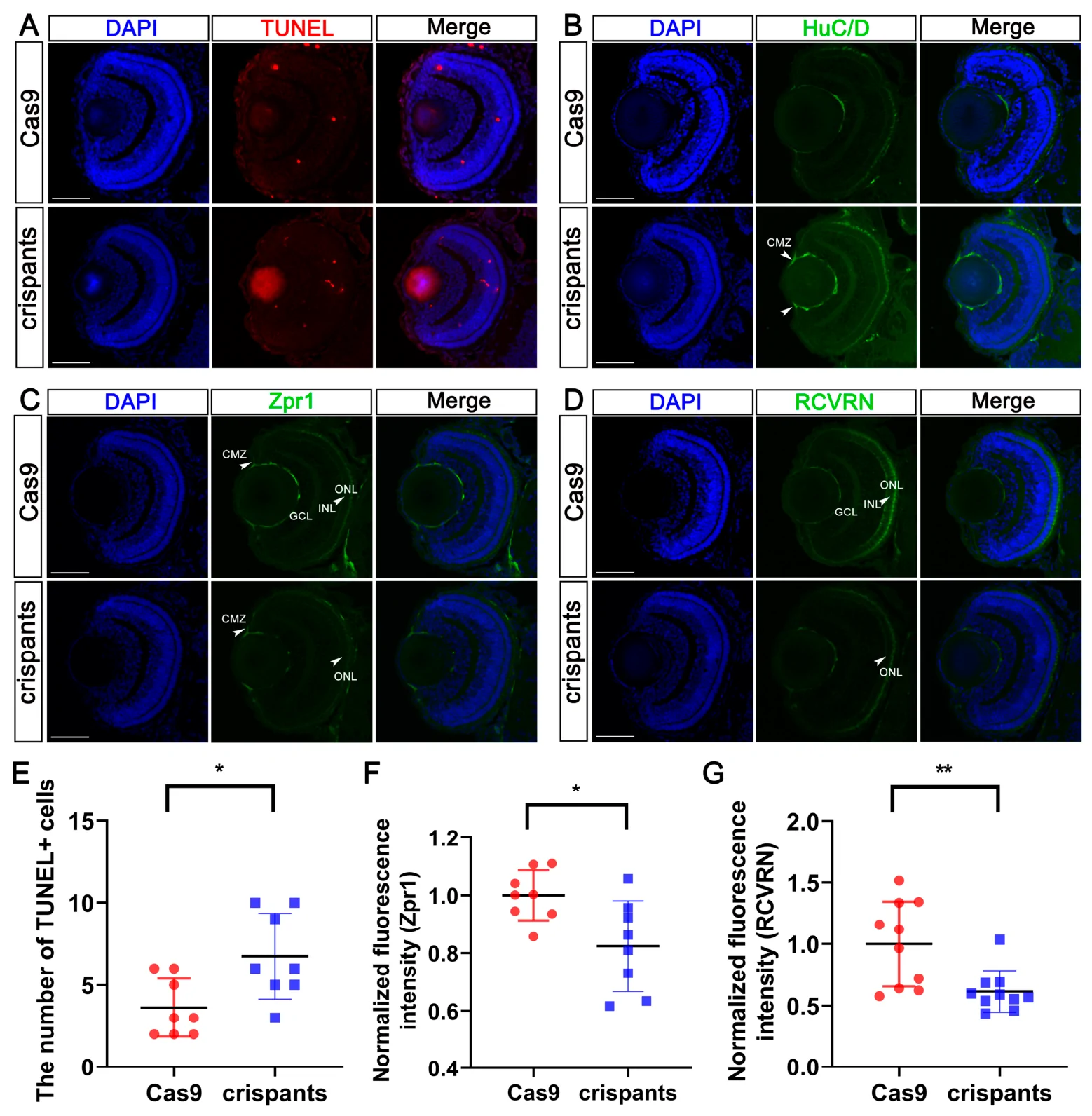
*mmp2* knockdown leads to retinal development impairment in zebrafish. Immunostaining for (**A**) TUNEL, (**B**) HuC/D, (**C**) Zpr1 and (**D**) RCVRN in *mmp2*-deficient and control AB zebrafish strains at 72 hpf. Nuclei were stained with DAPI (blue). TUNEL-positive cells were shown in red, while HuC/D, Zpr1, and RCVRN were shown in green. Scale bar = 50 μm. (**E**) Quantification of the number of TUNEL-positive apoptotic cells in each eye. Only cells with condensed apoptotic nuclei were counted. (**F**,**G**) Quantification of normalized mean fluorescence intensity of Zpr1 and RCVRN in the same area of the ONL. * *p* < 0.05, ** *p* < 0.01, versus control. *n* = 8/10 individual eyes per group.

To better elucidate the mechanisms underlying abnormal eye development following *mmp2* knockdown, we conducted cell apoptosis assays. TUNEL staining showed that *mmp2* deficiency led to a marked increase in apoptotic cells compared with the Cas9 control (Figure 3A,E). The concurrent increase in apoptosis may reflect a role for *mmp2* in tissue remodeling, and its disruption could cause structural disorganization.

Differentiated neurons were labeled with HuC/D. After *mmp2* deficiency, enhanced fluorescence signals were observed in the ciliary margin zone (CMZ) (Figure 3B). Notably, the CMZ is primarily composed of retinal progenitor cells and does not normally express the differentiation marker HuC/D. This ectopic expression may indicate a disruption of the differentiation process.

To further explore retinal structural changes in *mmp2*-deficient larvae, cone cells were examined using Zpr1, and photoreceptor cells using Recoverin (RCVRN). At 72 hpf, compared with Cas9 controls, the Zpr1 fluorescence signal was significantly weakened in *mmp2* crispants, and abnormal signals were also observed in the CMZ (Figure 3C,F). These findings indicate that *mmp2* knockdown impairs cone cell differentiation/maturation and is associated with abnormal distribution of Zpr1-positive cells in the CMZ. In addition, *mmp2* knockdown led to a significant reduction in RCVRN signals in photoreceptor cells (Figure 3D,G), suggesting decreased Recoverin expression and/or impaired photoreceptor differentiation/maturation. Together, these results show increased apoptosis and reduced Zpr1 and RCVRN immunofluorescence in *mmp2* crispants.

### 3.4. Transcriptomic Analysis Identifies Key Regulatory Genes and Pathways in Zebrafish Eye Development

**Figure 4:**
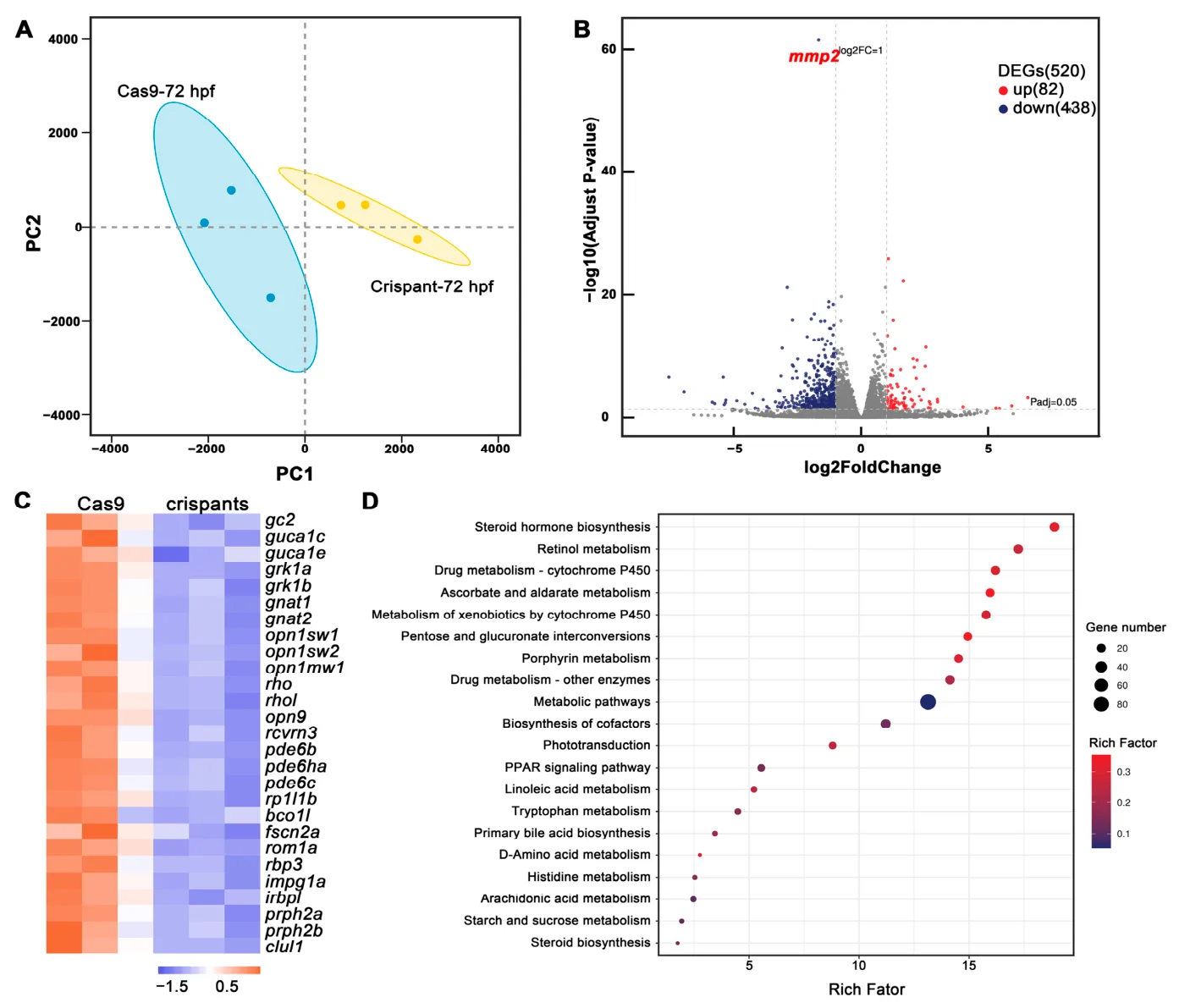
Transcriptional expression analysis of zebrafish embryos lacking *mmp2*. (**A**) PCA plot between Cas9 and crispant groups in zebrafish embryos. The blue points and ellipse represent the Cas9 group, while the yellow points and ellipse represent the *mmp2* crispants. (**B**) Volcano plot of DEGs between Cas9 and crispant groups in zebrafish embryos. Red dots indicate significantly upregulated genes, blue dots indicate significantly downregulated genes, and gray dots indicate genes with no significant difference. (**C**) The clustering heatmap of genes related to eye development. The color scale ranges from blue (low expression) to red (high expression). (**D**) KEGG enrichment analysis of up-/downregulated differential gene expression in different treatment groups. The size of the black dots represents the number of genes, and the color gradient from purple to red indicates the enrichment factor.

To reveal the potential molecular mechanism underlying *mmp2* deficiency during embryonic development, we performed transcriptome sequencing on zebrafish embryos at 72 hpf. Principal component analysis (PCA) distinctly separated the Cas9 and *mmp2* crispant groups (Figure 4A), indicating that *mmp2* deficiency alters the expression profile, and confirming the homogeneity of samples and experimental reliability. Differential expression analysis identified 520 differentially expressed genes (DEGs), of which 82 were upregulated and 438 were downregulated, with *mmp2* expression itself significantly decreased (Figure 4B), further validating the experimental model. A heatmap showed that all eye-development-related DEGs were downregulated (Figure 4C). KEGG analysis of the DEGs highlighted strong enrichment in the phototransduction and retinol metabolism pathways (Figure 4D).

Furthermore, we quantified the transcript levels of additional genes related to eye development through RT-qPCR. The retinal-related genes *ptf1a* and *grk1b* were significantly downregulated at both 48 and 72 hpf, *gfap* was downregulated at 48 hpf, and *atoh7* was downregulated at 72 hpf (Figure S4A). Lens-related genes *cryba1a* and *crygmx* were significantly decreased at both 48 and 72 hpf, whereas *cryba1b* was downregulated at 48 hpf but upregulated at 72 hpf. Additionally, *crygm2d2* was significantly downregulated only at 48 hpf (Figure S4B).

### 3.5. Metabolomic Changes and Integrated Analysis of Transcriptomic and Metabolomic Data in Zebrafish Eye Development

**Figure 5:**
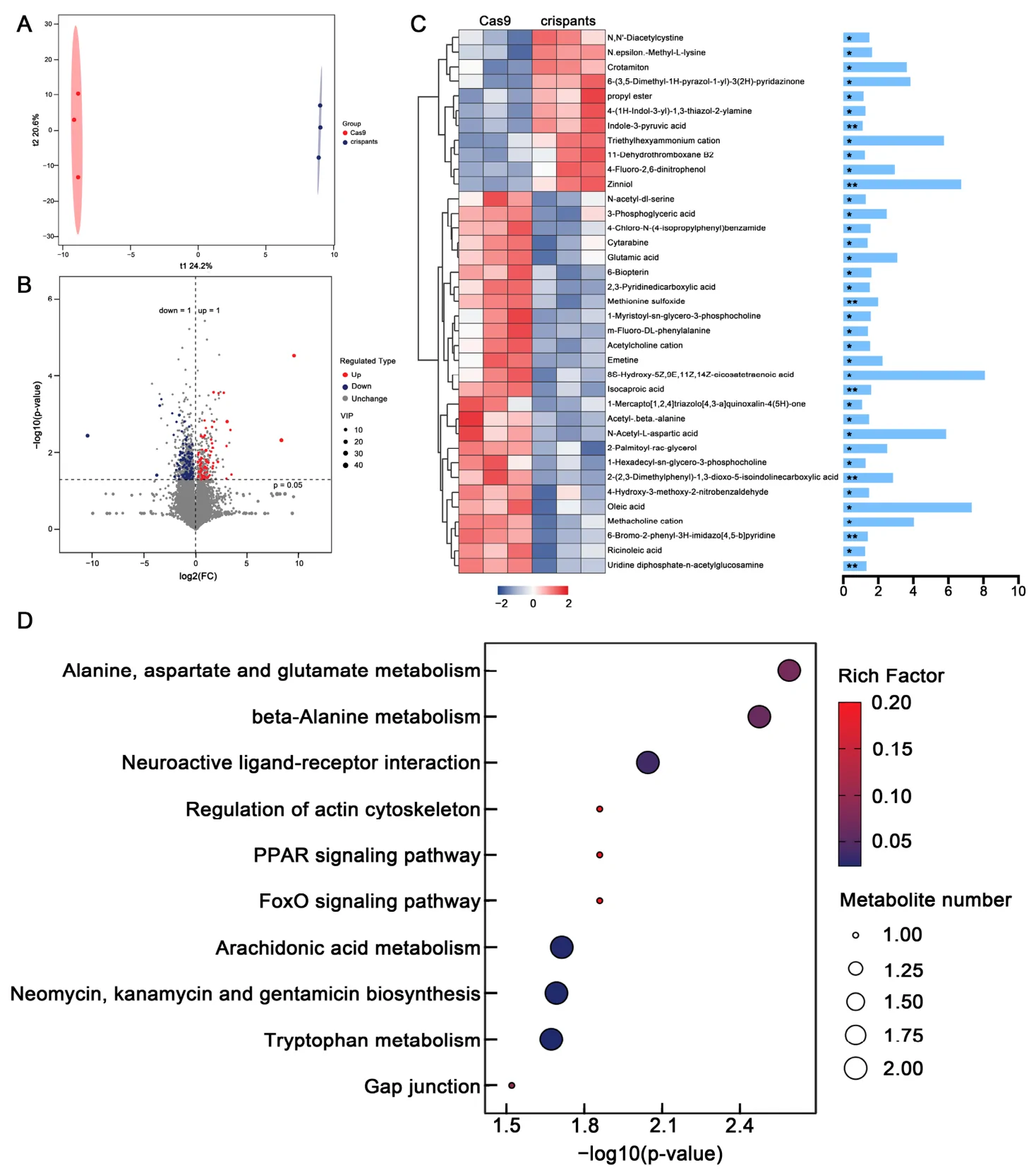
Metabolomics analysis of zebrafish embryos lacking *mmp2*. (**A**) OPLS-DA score plot shows the clustering and distribution of the Cas9 (red dots) and crispant (blue dots) groups. (**B**) The volcano plot represents the screening of differentially abundant metabolites. Red dots indicate upregulated metabolites, dark blue dots indicate downregulated metabolites, and gray dots indicate metabolites with no significant change. (**C**) The left figure is a heat map of hierarchical clustering analysis of DMs. The color scale ranges from blue (low relative abundance) to red (high relative abundance). The right figure shows the VIP values and *p*-values of metabolites. * *p* < 0.05, ** *p* < 0.01, versus control. (**D**) The bubble chart displays the enriched pathways in the Cas9 and crispant groups. The dot size represents the number of metabolites, and the color gradient from dark purple to red indicates the Rich Factor.

Untargeted metabolomics analysis was performed to examine the effects of *mmp2* deficiency. OPLS-DA revealed clustering of samples within the same treatment groups and a clear separation between the metabolic profiles of the Cas9 and crispant groups (Figure 5A). In comparison to the Cas9 group, 26 metabolites were found to be upregulated, while 11 were downregulated in the crispant samples. The most significantly up- and downregulated compounds were 4-fluoro-2,6-dinitrophenol and 1-mercapto [1,2,4]triazolo[4,3-a]quinoxalin-4(5H)-one, respectively (Figure 5B). Hierarchical clustering of the 37 differential metabolites is shown in the heatmap (Figure 5C). VIP (variable importance in projection) analysis was performed with a default threshold of VIP ≥ 1. The results showed that *mmp2* knockdown significantly reduced the levels of uridine diphosphate-N-acetylglucosamine, ricinoleic acid, oleic acid, and N-Acetyl-L-aspartic acid, while the level of 11-Dehydrothromboxane B2 was significantly increased (Figure 5C). Disruption of these nucleotides, lipids and amino acids may be related to the abnormal eye development caused by *mmp2* deficiency. Subsequently, KEGG enrichment analysis of the differential metabolites (DM) indicated that *mmp2* knockdown affected lipid metabolism (e.g., arachidonic acid metabolism) and amino acid metabolism (e.g., alanine, aspartate and glutamate metabolism, beta-alanine metabolism and tryptophan metabolism) (Figure 5D).

**Figure 6:**
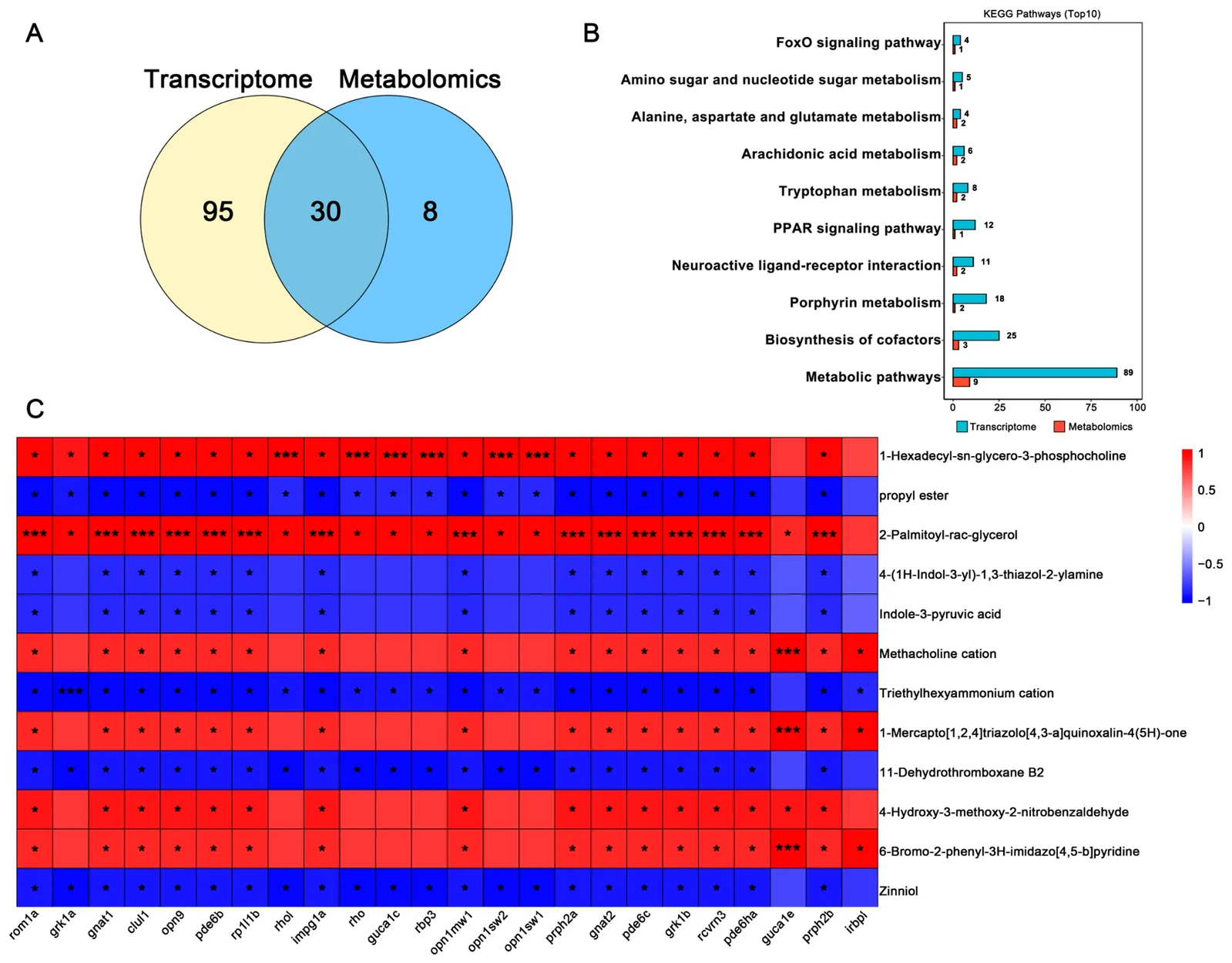
Joint analysis of the transcriptome and metabolome after *mmp2* knockdown. (**A**) The Venn diagram Venn diagram showing the overlap between transcriptome and metabolome data; yellow and blue circles represent the transcriptome and metabolome, respectively, with the overlap indicating shared items. (**B**) Bar graph illustrated KEGG pathways that are co-enriched in the transcriptome and the metabolome. The blue and red bars indicate the gene and metabolite numbers, respectively. (**C**) Correlation analysis between the DMs and the DEGs related to eye development. Red and blue respectively indicate positive and negative correlation. Colors range from blue (negative correlation) to red (positive correlation), with white (0) indicating no correlation. The asterisks denote statistical significance (* *p* < 0.05, *** *p* < 0.001). Unmarked boxes indicate non-significant correlations (*p* > 0.05).

We next performed an integrated analysis of DEGs and differential metabolites. The results showed 95 pathways unique to DEGs, 8 pathways unique to DMs, and 30 pathways shared between the two omics datasets (Figure 6A). The top 10 KEGG pathways with the largest number of co-participating molecules were visualized, including the PPAR signaling pathway, tryptophan metabolism, arachidonic acid metabolism, amino sugar and nucleotide sugar metabolism, alanine, aspartate and glutamate metabolism, etc (Figure 6B). To visually represent differences in expression patterns, Spearman correlation and hierarchical clustering analyses were performed between metabolites and transcriptional differences. The analysis showed that 24 eye-development-related genes were significantly correlated with 12 metabolites. Notably, *gnat1* and *gnat2* exhibited a highly significant positive correlation with 2-Palmitoyl-rac-glycerol, and *opn1sw1* and *opn1sw2* showed a highly significant positive correlation with 1-Hexadecyl-sn-glycero-3-phosphocholine, while *gnat1*, *gnat2*, *opn1sw1* and *opn1sw2* were all negatively correlated with 11-Dehydrothromboxane B2 (Figure 6C).

## 4. Discussion

Microphthalmia, defined as a congenitally small eye, represents a severe structural birth defect and a major cause of childhood blindness worldwide [1,30]. Although a growing number of genes have been linked to microphthalmia–anophthalmia–coloboma (MAC) spectrum disorders, they explain only a fraction of clinical cases, underscoring the need to identify novel regulators of ocular development [12,19,31,32]. In this study, we demonstrate that Mmp2 deficiency in zebrafish leads to a microphthalmia phenotype accompanied by disrupted retinal differentiation, impaired photoreceptor differentiation, and enhanced apoptosis, with multi-omic analyses further identifying dysregulated phototransduction, retinol metabolism and lipid metabolic pathways.

### 4.1. Mmp2 Function Within the Broader Mmp Family in Eye Development

Prior research has established a critical role for Mmps in vertebrate eye development [33]. Mmps are essential for maintaining and remodeling tissue architecture, with substrates that range from extracellular matrix (ECM) components to secreted cytokines and cell surface molecules [6,34,35]. In zebrafish, *mmp14a* deficiency disrupts retinoblast cell-cycle kinetics, delays retinal neurogenesis, and causes microphthalmia [12]. More directly, Mmp2 has been implicated in optic fissure fusion, where hyaloid vasculature-derived Mmp2 degrades the fissure basement membrane [12,19]. These reports collectively point to a developmentally essential role for certain MMPs in ocular morphogenesis. Our data extend these observations by demonstrating that Mmp2 loss in zebrafish embryos causes microphthalmia. This pattern of differentiation defects suggests that Mmp2 may be required not only for early morphogenetic events such as fissure closure but also for the later establishment of proper retinal layer architecture.

### 4.2. Concurrent Apoptosis and Proliferation: A Compensatory or Disruptive Mechanism?

At the cellular level, our results reveal a complex phenotype characterized by enhanced retinal apoptosis (demonstrated by TUNEL). Janssens et al. established that Mmp14a, the principal physiological activator of pro-Mmp2, regulates retinoblast cell-cycle kinetics and thereby the timing of retinal neurogenesis, and reported a possible co-involvement of Mmp2 in retinotectal development [12]. Weaver et al. demonstrated that Mmp2 activity is required for degradation of the optic fissure basement membrane during fusion [19]. Neither study, however, examined whether Mmp2 continues to influence cell survival and cell-cycle behavior after the optic fissure has closed and retinal differentiation is underway. Our data address precisely this window: at 72 hpf, *mmp2* knockdown produces the concurrent apoptotic and proliferative phenotype described here, which is not predicted by, and cannot be inferred from, the optic fissure fusion or early neurogenesis functions previously assigned to this protease.

A similar dissociation between apoptosis and eye size has been reported in other zebrafish microphthalmia models, and it supports the interpretation that the two cellular processes are not simply coupled. In *gdf6a* deficient zebrafish, ectopic retinal apoptosis was observed, yet anti-apoptotic rescue did not restore normal eye size, indicating that microphthalmia arises from disruption of multiple developmental processes rather than from cell death alone [36]. Likewise, *flrt2* knockout zebrafish exhibit increased apoptosis in retinal progenitor cells, but retinal progenitor proliferation and differentiation are not affected, and the microphthalmia is attributed to the combined dysregulation of *crx*, *neurod4*, *atoh7* and caspase genes [37]. These examples establish that apoptosis and proliferation can be independently perturbed in the developing retina. In the *mmp2* crispant, the simultaneous increase in apoptosis and altered proliferation is therefore best interpreted not as a single linear consequence of cell loss, but as a coordinated disruption of the ECM microenvironment that normally constrains both survival and cell-cycle entry.

This interpretation is consistent with what is known about how gelatinases influence retinal proliferation. In the chick retina, inhibiting Mmp2 prior to damage or growth-factor treatment potentiates the proliferative response, whereas selective Mmp9 inhibition has no such effect, implicating Mmp2 specifically in suppressing progenitor expansion under certain conditions [16]. In zebrafish, Mmp2/Mmp9 activity is required for the efficiency of regeneration-associated genes in generating Müller glia-derived progenitor cells, and pharmacological blockade of these gelatinases reduces progenitor numbers [38]. Mmp9 loss-of-function, by contrast, causes hyperproliferation of Müller glia-derived progenitors, indicating that distinct gelatinases can exert opposing effects on retinal progenitor behavior [39]. The *mmp2* knockdown phenotype reported here therefore fits a model in which Mmp2 is not a generic permissive factor for proliferation, but a context-dependent modulator of the balance between progenitor maintenance and cell-cycle exit, a function that would be invisible in assays focused exclusively on optic fissure fusion. MMP9 has been shown to compromise cell–cell adhesion and signaling events through actions on ECM substrates [40], and MMP13 activity influences cell proliferation and cell-cycle progression in melanocytes and melanoma cells [41]. In our model, the simultaneous increase in apoptosis may reflect a compensatory response to tissue disorganization: the retina attempts to replace lost cells but fails to do so in a coordinated manner, ultimately leading to net structural deficits. Alternatively, Mmp2 deficiency may disrupt the balance between ECM degradation and deposition, thereby altering the microenvironment for both cell survival and cell-cycle regulation.

### 4.3. Mmp2 Is Required for Photoreceptor Differentiation and Survival

The observed reduction in Zpr1 and Recoverin signals constitutes one of the most striking features of the *mmp2* crispant phenotype; it defines the principal conceptual advance of this study relative to previous reports. Immunostaining for Zpr1 (cone cell marker) and Recoverin (photoreceptor marker) revealed significantly diminished signals at 72 hpf. Janssens et al. documented *mmp2* expression in the retina from 24 hpf onwards, and Weaver et al. showed that *mmp2* is functionally necessary for optic fissure basement membrane degradation [12,19]. Neither study, however, assessed whether Mmp2 is required for the acquisition or maintenance of photoreceptor identity after the fissure has fused. The diminished Zpr1 and Recoverin signals, together with the reduced expression of cone-opsin, rhodopsin, establish that Mmp2 function extends beyond morphogenesis into the differentiation program of post-mitotic photoreceptors. This is a distinct function, not a downstream consequence of fissure fusion failure. Because the CRISPR/Cas9 reagents were injected during early embryogenesis, Mmp2 perturbation was initiated at an early developmental stage rather than at 72 hpf. We therefore cannot exclude the possibility that the retinal abnormalities observed at later stages arise, at least in part, from earlier developmental defects.

This finding is consistent with the emerging view that Mmp2 is not merely an ECM remodeling enzyme but also plays a role in preventing the death of photoreceptor cells in retina. Although photoreceptor survival was not directly assessed, the observed changes may reflect impaired photoreceptor maintenance in retina after Mmp2 perturbation. Indeed, in experimental retinal detachment, Mmp2 activation coincides with photoreceptor cell death, and modulation of Mmp2 activity influences photoreceptor integration in degenerative retina models [42]. Beyond pathological contexts, during retinal regeneration in zebrafish, *mmp2* expression is strongly upregulated at the time when neuronal progenitor cells begin migrating to the outer nuclear layer [43], suggesting that Mmp2 may facilitate the migration and differentiation of newly generated photoreceptors. In our study, the impaired photoreceptor differentiation combined with ectopic expression of cone markers in the CMZ, a region normally reserved for retinal progenitor cells that do not express the differentiation marker HuC/D, suggests that Mmp2 deficiency disrupts the timing and spatial coordination of retinal cell fate specification, leading to both differentiation failure and mislocalization.

It is important to note that Mmp2 has established roles in early ocular morphogenesis and extracellular matrix remodeling [12,19]. Consequently, the alterations in photoreceptor markers and retinal organization observed here may reflect a specific requirement for Mmp2 in photoreceptor differentiation and/or secondary consequences of earlier developmental defects. The present study cannot definitively distinguish between these possibilities. Future conditional or temporal-specific Mmp2 ablation/rescue experiments will be required to determine whether Mmp2 acts directly within differentiating photoreceptors or indirectly through remodeling of the retinal microenvironment.

The reduced Zpr1 and Recoverin immunofluorescence observed here is indicative of altered photoreceptor marker expression and impaired photoreceptor differentiation, but it does not by itself demonstrate a reduction in photoreceptor cell numbers. Because these markers reflect differentiation status rather than cell survival, the present data cannot distinguish between reduced marker expression per cell, delayed or impaired differentiation, and actual photoreceptor loss. Definitive discrimination between these possibilities would require unbiased stereological counts and co-labeling with cell-death or lineage markers, which we identify as an important direction for future work.

### 4.4. Transcriptomic Insights: Phototransduction and Retinol Metabolism Pathways

The transcriptomic analysis performed at 72 hpf provides molecular level insight into the mechanisms underlying the observed phenotypes. Principal component analysis (PCA) clearly separated the Cas9 control and *mmp2* crispant groups, confirming that Mmp2 loss profoundly alters the retinal expression program. Among the 520 DEGs identified, the vast majority (438 genes) were downregulated, indicating that Mmp2 may be required for the proper expression of a large set of genes, particularly those involved in phototransduction and retinol metabolism pathways. KEGG enrichment analysis highlighted phototransduction as a top dysregulated pathway [28], a finding that resonates with previous reports in which disruption of phototransduction was associated with abnormal ocular morphology [44]. The downregulation of *gnat1*, *gnat2*, *opn1sw1*, *opn1sw2*, and *rho* in our RT-qPCR data further substantiates this pathway level defect. Interestingly, while WISH revealed enhanced signals of certain genes at 72 hpf, RT-qPCR showed downregulation for the same genes. We hypothesize that this discrepancy may stem from altered spatial distribution of transcripts; WISH captures both transcript abundance and localization, and ectopic expression in regions such as the CMZ could contribute to a perceived increase in signal intensity despite a global reduction in transcript levels as measured by RT-qPCR. However, this remains a hypothesis and cannot be definitively concluded from the current data; further single-cell or spatial transcriptomic analyses would be required to verify the precise cellular localization and expression levels of these transcripts.

Because the molecular analyses in this study were performed on whole embryos rather than isolated ocular tissues, the observed alterations may reflect a combination of retinal and non-retinal developmental effects. Accordingly, the present data should not be interpreted as establishing retina-specific molecular mechanisms. Rather, they identify molecular changes associated with Mmp2 perturbation that may contribute to the retinal phenotype. Future studies using isolated ocular tissues, retinal cell-type-specific profiling, or conditional Mmp2 perturbation will be necessary to distinguish retina-intrinsic mechanisms from broader developmental consequences.

### 4.5. Metabolomic Alterations and Integrated Omics Analysis

The metabolomic analysis revealed that Mmp2 deficiency leads to significant alterations in multiple metabolic pathways, including arachidonic acid metabolism, tryptophan metabolism, and alanine, aspartate and glutamate metabolism. Metabolomic profiling has been increasingly applied in zebrafish models to uncover perturbations in lipid, nucleotide and amino acid metabolism associated with ocular abnormalities [45]. Lipid metabolism disruption has been previously linked to microphthalmia in other contexts. For instance, early retinoic acid deprivation in developing zebrafish, which alters lipoprotein expression and lipid transport, results in microphthalmia [12,19,46]. Similarly, *cyp1b1* knockout zebrafish exhibit microphthalmia in association with dysregulated expression of lipid metabolism genes [45,47]. More recent metabolomic profiling of *cyp1b1* knockout zebrafish has revealed alterations in KEGG pathways associated with lipid, nucleotide, and amino acid metabolism [45], closely mirroring our observations in *mmp2* crispants. The significant reduction in oleic acid, ricinoleic acid and UDP-N-acetylglucosamine levels observed in our *mmp2* crispants further supports the notion that Mmp2 influences lipid metabolic homeostasis during eye development. These findings raise the possibility that Mmp2, through its proteolytic activity, may regulate the bioavailability of signaling lipids or influence the activity of enzymes involved in lipid metabolism, thereby affecting retinal development. The integrated transcriptomic and metabolomic analysis revealed 30 shared pathways, including the PPAR signaling pathway, which is known to regulate lipid metabolism and has been implicated in retinal development [48,49,50,51] and disease, including retinal degeneration [52], diabetic microvascular damage [53], diabetic retinopathy (DR) [54]. The strong correlations between specific eye-development-related genes and metabolites, such as the positive correlations of *gnat1* and *gnat2* with 2-palmitoyl-rac-glycerol, and the negative correlation of these same genes with 11-dehydrothromboxane B2, highlight the intimate coupling between gene expression and metabolic state in the developing eye.

## 5. Limitations of the Study

Several methodological limitations deserve mention. First, we employed a crispant approach rather than a stable *mmp2* knockout line. While crispants enable rapid phenotypic assessment, they may exhibit interindividual variability in knockdown efficiency and potential off target effects. However, the F0 CRISPR-Cas9 knockout approach has been demonstrated to effectively recapitulate gene functions at least until 5 dpf in zebrafish [55]. The future generation of a stable *mmp2* null mutant line would provide a more definitive assessment of the gene’s role in eye development. Second, the precise molecular mechanisms through which Mmp2 regulates photoreceptor differentiation and survival have yet to be fully elucidated. Although we have identified multiple dysregulated pathways, whether these changes are direct consequences of Mmp2 proteolytic activity or secondary effects of broader tissue disorganization requires further investigation using cell-type-specific manipulations. Third, our omic analyses were performed on whole embryos, which, while informative for global pathway identification, lack cellular resolution. Single-cell transcriptomic and metabolomic approaches in future studies would help pinpoint the specific retinal cell types primarily affected by Mmp2 loss.

## 6. Conclusions

In summary, this study demonstrates that Mmp2 is essential for normal eye development in zebrafish, acting at multiple levels: maintaining proper retinal differentiation, promoting photoreceptor differentiation and survival, and regulating the expression of key phototransduction and metabolic pathways. Loss of Mmp2 disrupts these processes. By integrating morphological, cellular, transcriptomic and metabolomic data, we provide a comprehensive view of the consequences of Mmp2 deficiency in the developing eye. Given the evolutionary conservation of Mmp2 function and retinal architecture between zebrafish and humans, our findings may have relevance for understanding the molecular basis of microphthalmia and other ocular developmental anomalies in humans.

## Data Availability

All the original data presented in this study are included in the article and Supplementary Materials. For further inquiries, please contact the corresponding author. The original data of transcriptomics and metabolomics in this article are openly available in NCBI (PRJNA1484010) and MetaboLights (MTBLS15010).

## Supplementary Materials

The following supporting information can be downloaded at: https://www.mdpi.com/article/10.3390/cells15211993/s1, Table S1: The primers used for WISH in this study; Table S2: The primers used for qRT-PCR in this study; Figure S1: The spatial expression pattern of mmp2 in zebrafish; Figure S2: Generation of mmp2 crispants in zebrafish using CRISPR/Cas9; Figure S3: WISH and RT-qRCR analysis of eye-related genes in mmp2 deficient embryos; Figure S4: Gene expression levels in zebrafish embryos in mmp2 deficiency at 48 and 72 hpf.
