# Supplementary material for: Matrix Metalloproteinase-2 Deficiency Impairs Eye Development in Crispant Zebrafish
Source: Cells. 2026 Oct 31;15(21):1993. doi: 10.3390/cells15211993 (PMC13762441; doi:10.3390/cells15211993)
Supplement: Supplementary file 1 [file cells-15-01993-s001.zip › cells-4636751-supplementary.pdf]

# Supplementary Materials

# Matrix Metalloproteinase-2 Deficiency Impairs Eye Development in Crispant Zebrafish

**Chenhui Ren and Lu Wei ***

Hainan Province Key Laboratory of One Health, School of Life and Health Sciences, Collaborative Innovation Center of Life and Health, Hainan University, Haikou 570228, China
* Correspondence: luwei@hainanu.edu.cn

## Contents of Supporting Information

**Supplementary materials and methods**

**Table S1** The primers used for WISH in this study.

**Table S2** The primers used for qRT-PCR in this study.

**Figure S1**. The spatial expression pattern of *mmp2* in zebrafish.

**Figure S2**. Generation of *mmp2* crispants in zebrafish using CRISPR/Cas9.

**Figure S3**. WISH and RT-qRCR analysis of eye-related genes in *mmp2* deficient embryos.

**Figure S4**. Gene expression levels in zebrafish embryos in *mmp2* deficiency at 48 and 72 hpf.

Table S1. The primers used for WISH in this study.

| Gene name | Primer (5' - 3') |
|---|---|
| *gnat1*-F | GAGTGCTTGGAGTTCATTGTC |
| *gnat1*-R | ATCCACTTCTTCCTCTCTGAG |
| *gnat2*-F | ATGTTTGATGTGGGTGGC |
| *gnat2*-R | ATGGAAGTTGTGGCGAAG |
| *opn1sw1*-F | TTTGGATGGAGCAGATAC |
| *opn1sw1*-R | TGAGAAGAAAGCAGGGAT |
| *opn1sw2*-F | CCTCGGGAACTTTACCT |
| *opn1sw2*-R | TTGGAAACCACCCAGATT |
| *opn1lw1*-F | TGAGGGTCCCAATTACCA |
| *opn1lw1*-R | AGCAGATGCCCATTTAGC |
| *rhodopsin*-F | CTACGTGCCTATGTCCAATGCCAC |
| *rhodopsin*-R | CAGACCACCATCCAGCGCTCAAT |
| *vsx2*-F | CTGTCAAAGAGCAGCATCACAC |
| *vsx2*-R | CTACTGCGACCCCAACACTTC |
| *mmp2*-F | TGATGATGATGGAAAGTACGGCT |
| *mmp2*-R | CAATGGCTTGTCTGTTGGTTCTC |

Table S2. The primers used for qRT-PCR in this study.

| Gene name | Forward Primer (5' - 3') | Reverse Primer (5' - 3') |
|---|---|---|
| *opn1sw1* | CTTTGCTATGCCCCTTATGC | ATGAGGTTTCGGTCTTGCTG |
| *opn1sw2* | CCTCGGGAACTTTACCTT | TTGGAAACCACCCAGATT |
| *opn1mw1* | ACAGCCCAGCACAAGAAACT | AGCCTAATGGACCAAGAGCC |
| *opn1lw1* | TAGCACTGCTTGAGGGAATGT | GGGCAGGCATCTACCTATCAC |
| *rhodopsin* | CCTGCCTCGTTTCTTCACA | TGGGTATTCGTATGGGCTC |
| *gnat1* | AGGATTCAGGCATCCAGGCG | CCGGGCTGGATCAACCTCTC |
| *gnat2* | CGACGAGATGGGTAGCGGAGC | GCCCCTGATGATGGCCAGAGC |
| *vsx2* | GAGCAGCATCACACATCCGCC | AACCTCCAGAAACGCGGGCT |
| *ptf1a* | CCCAGACTACGGGTTGCCTC | GTCCAGACTTTCGCTGTCCG |
| *gfap* | ACTGAGGAGTGGTATCGCTCAAA | AGACCCACGGAGAGATTCCA |
| *atoh7* | CCTGAACACGGCATTCGACC | GCGTCGCTCAGGATCCGATT |
| *grk1b* | CAGGTGGACAGCAATGAA | AAAGGTGGAGGAAGCATAC |
| *crybgx* | GCTCAGGGTGACCACATCTC | CTGATACGCCACCCAGGTTC |
| *crygm2c* | CCATGCACGGAAAGGTCATC | GCATGGGAATCATTCGGCAA |
| *cryba1a* | TTGCAGCTTTTTGTTGGGTCT | ATTGGCCGGAAGGACATGAG |
| *cryba1b* | CAACAACGCCTATCGCATCG | CTGGTAGCATACAAAAGCGCC |
| *crygm2d2* | AACCATGATGGGCAAGGTCA | GGCAGGACCTGATGCAGTTG |
| *crygmx* | CGTGGTTCCTGCTTACACTGG | TGGAGGATCCAGTAGCCCTC |
| *mmp2* | AGATCTTCTTCTTTAAGGACAG | TACCCACATCTCATCACCAGC |
| *β-actin* | ATGGATGAGGAAATCGCTGCC | CTCCCTGATGTCTGGGTCGTC |

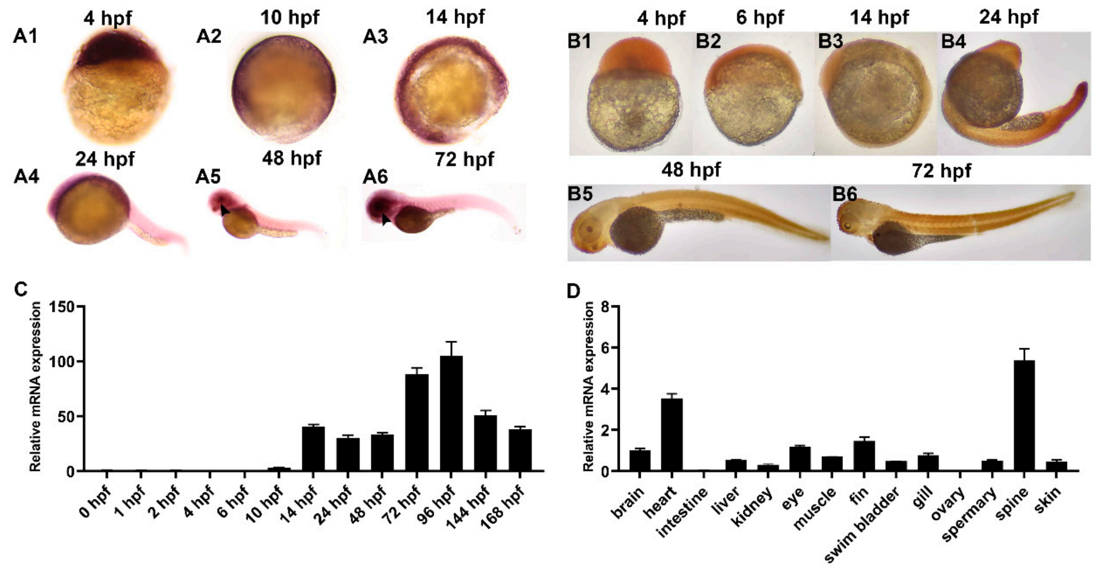


**Figure S1**. The spatial expression pattern of *mmp2* in zebrafish.

(A) Whole *in situ* hybridization (WISH) and (B) immunohistochemistry (IHC) show the localization of *mmp2* in zebrafish embryos at 4-72 hpf. qRT-PCR detect the changes in the expression level of *mmp2* in (C) zebrafish embryos (D) and adult tissues.

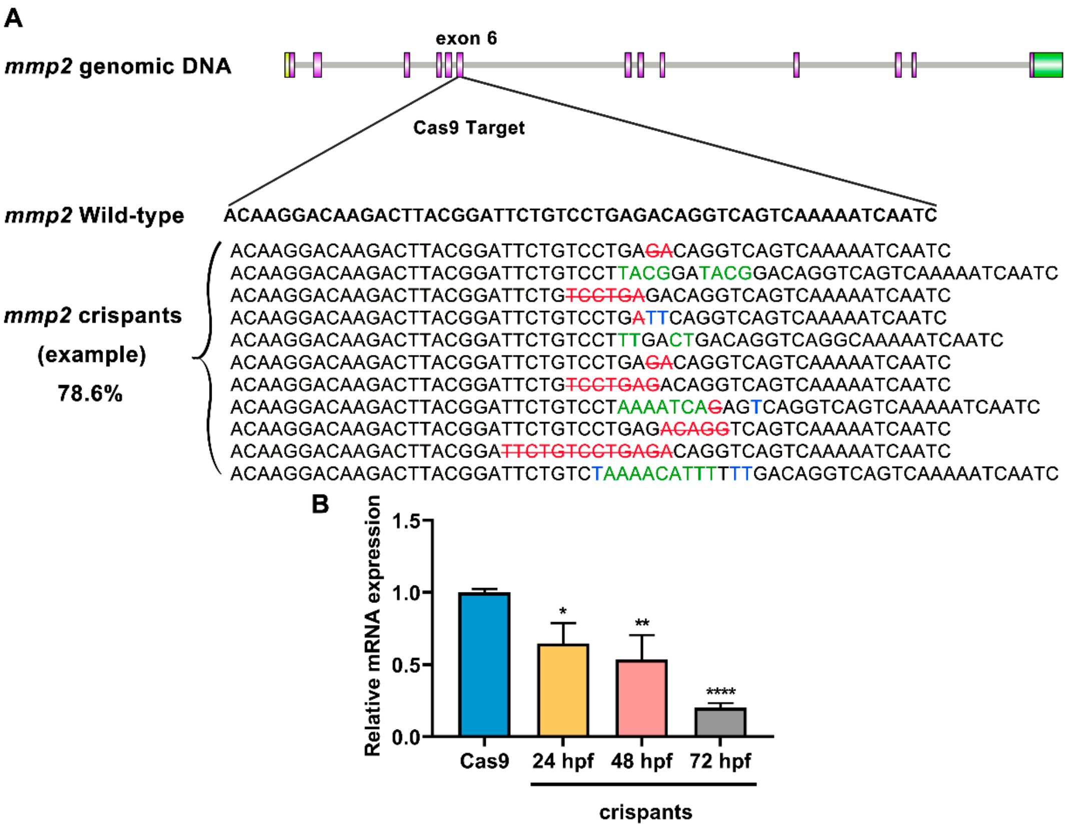


**Figure S2**. Generation of *mmp2* crispants in zebrafish using CRISPR/Cas9.

(A) The schematic of *mmp2* gene structure (top), wild-type zebrafish (middle), and mutant *mmp2* (bottom). (B) Quantification of mmp2 expression in *mmp2* crispant embryos.

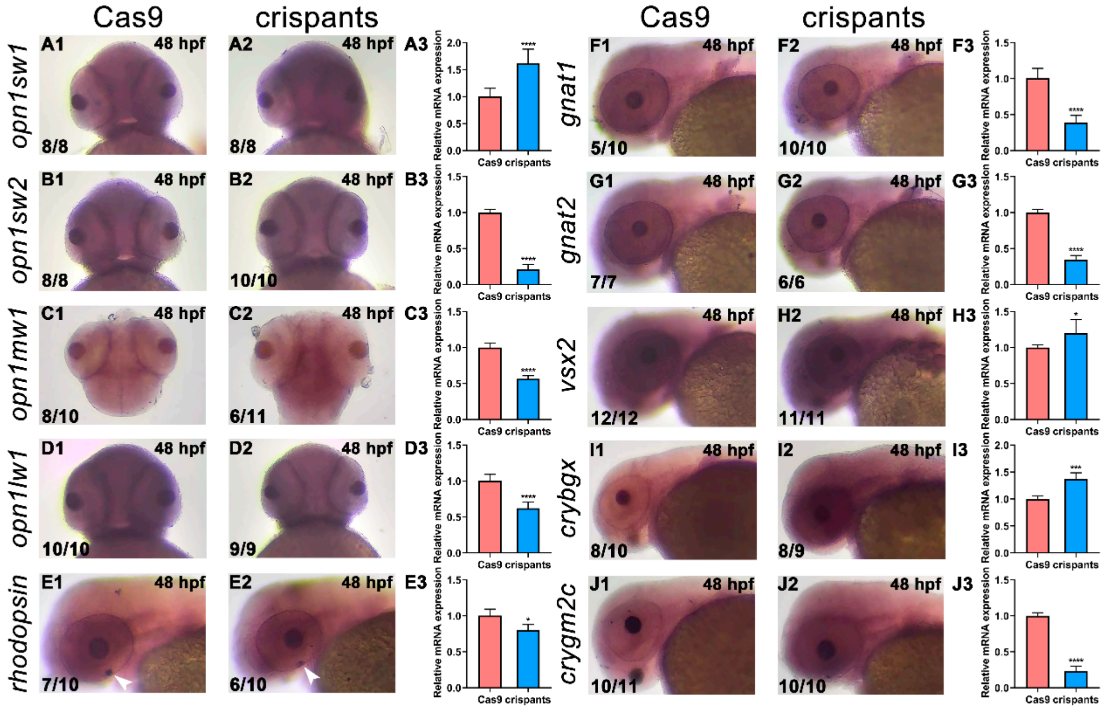


**Figure S3**. WISH and RT-qRCR analysis of eye-related genes in *mmp2* deficient embryos.

Expression patterns of (A) *opn1sw1*, (B) *opn1sw2*, (C) *opn1mw1*, (D), *opn1lw1*, (E) *rhodopsin*, (F) *gnat1*, (G) *gnat2*, (H) *vsx2*, (I) *crybgx* and (J) *crygm2c* in zebrafish embryos at 48 hpf.

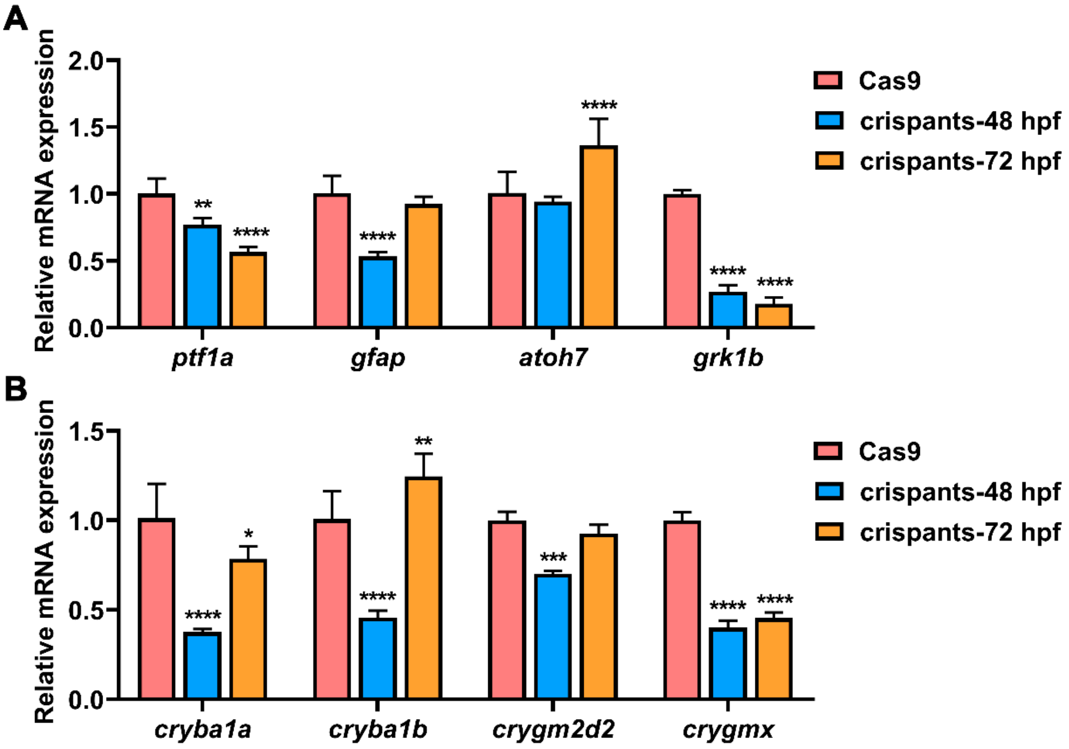


**Figure S4**. Gene expression levels in zebrafish embryos in *mmp2* deficiency at 48 and 72 hpf.

The expression levels of eye development-related genes of (A) Retinal cell type-specific genes (*ptf1a*, *gfap*, *atoh7* and *grk1b*), (B) Crystallin-β genes (*cryba1a*, *cryba1b*) and Crystallin-γ genes (*crygm2d2*, *crygmx*) were measured after *mmp2* knockdown.
